# Case of an Adult Never Furnished with Teeth

**Published:** 1859-03

**Authors:** A. Berry


### CASE OF AN ADULT NEVER FURNISHED WITH TEETH.

A negro man, aged about forty-five years, belonging to Col. W. H. H. of Claiborne county, Mississippi, with whom he has been during the last ten years, says that he never had any teeth.

I examined his mouth a few days ago, and found the alveolar ridges less developed, and the gums over them, not much, if any, firmer than is common with edentated persons; but the inferior alveolus, although very low, is broader than usual, and meets the superior very fairly for a considerable part of its anterior portion, enabling its possessor, as he says, (which is confirmed by others), to masticate readily any kind of food, even crust of bread. But he ought, undoubtedly, to adopt the first bill of fare, recorded—Genesis, first chapter, 26th verse,—and eschew all animal food.

His face presents a singular shortened appearance, and he keeps his inferior maxillary in motion a great part of the time. He is healthy; and I have no doubt of the truth of the report as to his want of teeth.

Jan. 24, 1859. A. Berry, D. D. S.

